# A Case of Adrenal Insufficiency Secondary to Bilateral Adrenal Hemorrhage in a Patient With Antiphospholipid Antibody Syndrome and Epstein-Barr Virus Infection

**DOI:** 10.7759/cureus.63544

**Published:** 2024-06-30

**Authors:** Grishma Pokharel, Stephanie Rosales, Jill Morrison, Christian Kim

**Affiliations:** 1 Internal Medicine, Englewood Hospital and Medical Center, Englewood, USA; 2 Hematology and Oncology, Englewood Hospital and Medical Center, Englewood, USA; 3 Endocrinology, Hackensack University and Medical Center, Hackensack, USA

**Keywords:** bilateral adrenal mass, adrenal insufficiency, antiphospholipid antibody, ebv infection, bilateral adrenal hemorrhage

## Abstract

Bilateral adrenal hemorrhage (AH) is linked to various causes, including bacterial and viral infections, coagulopathies, and postoperative states. Symptoms can range from mild adrenal insufficiency to shock from Waterhouse-Friedrichsen syndrome. We present a case of a 47-year-old male with antiphospholipid antibody syndrome (APS) on warfarin who presented to the emergency department (ED) with bilateral flank pain and was found to have bilateral AH. On exam, he was hypertensive, mildly tachycardic, and in severe pain. The abdomen was tender over the bilateral flank and costovertebral regions. Labs showed thrombocytopenia but normal international normalized ratio (INR) and fibrinogen. The CT and MRI confirmed bilateral AH. Further investigations revealed low ante meridiem (AM) cortisol and elevated adrenocorticotropic hormone (ACTH). The antinuclear antibody (ANA) test was negative, but the antiphospholipid antibody panel was positive. In addition, the patient had a positive Epstein-Barr virus (EBV) nuclear antigen with a significant IgM titer. He was treated with low-dose steroids and was placed on a prophylactic dose of enoxaparin with the resolution of symptoms. At discharge, he was advised to follow up with a hematologist in six weeks to restart full-dose anticoagulation, allowing time for the bleeding to resolve. This case highlights EBV infection as a possible trigger of adrenal insufficiency from adrenal bleeding in a patient with preexisting coagulopathy, necessitating prompt recognition and treatment.

## Introduction

Bilateral adrenal hemorrhage (AH) is a rare condition that can be related to various causes, such as systemic bacterial and viral infections, coagulopathies, and postoperative states [1,2]. Any stressful condition can induce physiological hyperplasia of the adrenal glands, leading to increased vascularity and a predisposition to bleeding. Diagnosing AH is challenging due to its often nonspecific signs and symptoms [3]. Clinical manifestations can vary from mild adrenal insufficiency to shock from Waterhouse-Friedrichsen syndrome, necessitating a high level of suspicion, prompt evaluation, and timely treatment. In the case below, we present how a middle-aged man with a history of antiphospholipid antibody syndrome (APS) on chronic warfarin presented with bilateral flank pain and was found to have bilateral AH in the setting of an Epstien-Barr virus (EBV) infection. 

## Case presentation

A 47-year-old male with APS on warfarin and a history of deep vein thrombosis and pulmonary embolism presented to the emergency department (ED) with a complaint of shifting pain in his bilateral flank, lower back, and chest for two days. At an outside hospital, a CT scan of his abdomen and pelvis revealed bilateral AH. He was told to stop warfarin and take an enoxaparin prophylactic dose daily, given the high risk of thrombosis due to APS and a history of pulmonary embolism in the past. One day later, he subsequently flew to our facility and came to the ED for worsening pain. On exam, he was hypertensive with a blood pressure of 140/90 mm/hg in the setting of severe pain (10/10) and mildly tachycardic to 110 beats/min. The abdomen was tender over the bilateral flank area and costovertebral region. Labs were remarkable for thrombocytopenia, with a platelet count of 51 k/uL, an international normalized ratio (INR) of 1.3, activated partial thromboplastin time (aPTT) of 78 seconds, and fibrinogen of 549 mg/dl, as represented in Table 1. 

**Table 1:** Laboratory results INR: international normalized ratio; aPTT: activated partial thromboplastin time; EBV: Epstein-Barr virus; VCA: viral capsid antigen; Ig: immunoglobulin; AM: ante meridiem; ACTH: adrenocorticotropic hormone; DHEA: dehydroepiandrosterone; ACE: angiotensin-converting enzyme

Laboratory parameter	Value	Reference range
Platelet	51 k/ul	150-400 k/ul
INR	1.3	0.9-1.1
aPTT	78 seconds	30-40 secs
Fibrinogen	549 mg/dl	200-400 mg/dl
Antiphospholipid antibody panel	Positive	Negative
EBV nuclear antigen	>600 U/ml	<18 U/ml
EBV VCA IGM titer	>160 U/ml	<36 U/ml
Antithrombin III activity	116%	80-135%
Factor V Leiden	Negative	Negative
Heparin-induced platelet antibody	Negative	Negative
AM cortisol	2.3 ug/dl	3.7-19.4 ug/dl
ACTH	850 pg/ml	6-50 pg/ml
Renin	2.60 ng/ml/hr	0.25-5.82 ng/ml/hr
Aldosterone	4 ng/dl	3.1-35.4 ng/dl
DHEA	156 mcg/dl	93-415 mcg/dl
ACE	40 U/l	9-67U/l
Metanephrines, 24-hour urine	240 mcg/24 hr	58-203 mcg/24 hour
Sodium	133 mg/dl	135-145 mg/dl
Potassium	4 mEq/l	3.5 to 5.2 mEq/l
Glucose	110 mg/dl	75-150 mg/dl

**Figure 1:**
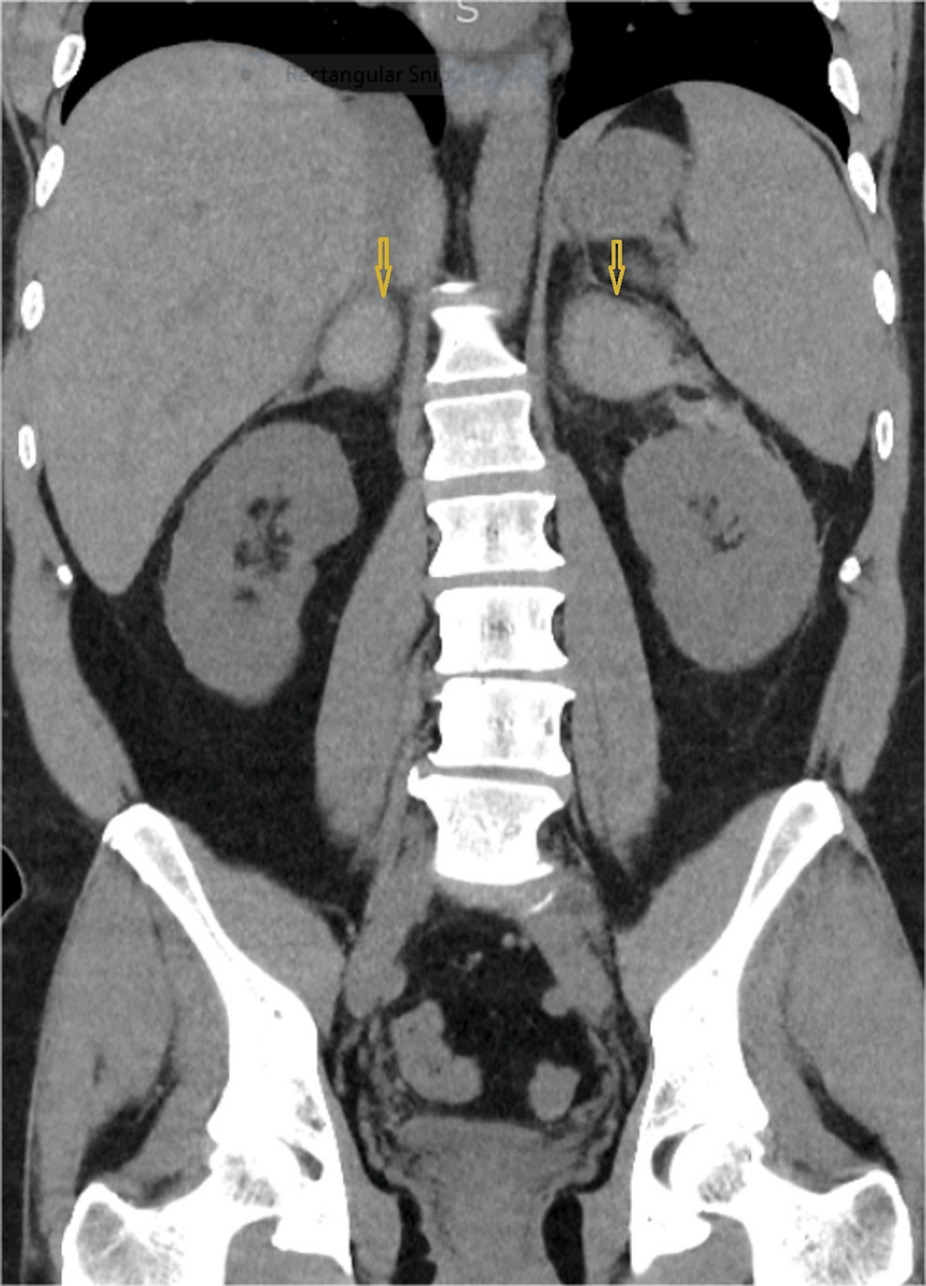
A longitudinal view of the CT scan of the abdomen and pelvis shows bilaterally enlarged adrenal glands (left>right) with adjacent hematoma shown by arrow

**Figure 2:**
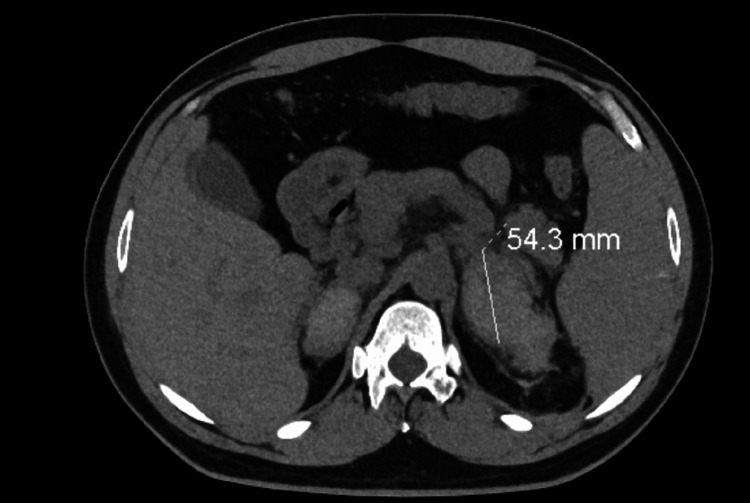
A transverse view of a non-contrast CT scan of the abdomen and pelvis shows a large left-sided adrenal hemorrhage.

A non-contrast CT scan of his abdomen and pelvis (Figures 1-2) showed bilateral AH. 

He was monitored in the ICU for the first 24 hours due to the risk of Waterhouse-Friedrichsen syndrome and a florid adrenal crisis. However, his pain was controlled, and he remained normotensive due to the prompt initiation of hydrocortisone. Further investigation showed a low ante meridiem (AM) cortisol level of 2.3 mcg/dl. Adrenocorticotrophic hormone (ACTH) was elevated at 850 pg/ml (normal range: 6-50 pg/ml). Renin, aldosterone, dehydroepiandosterone (DHEAS), and angiotensin-converting enzyme (ACE) were within normal limits. Antithrombin III activity was within the normal range. No antiplatelet antibodies against heparin were detected. The antinuclear antibody (ANA) test was negative. The antiphospholipid antibody panel was positive for B2 glycoprotein I Ab IgA and IgG, phosphatidylserine/prothrombin Ab IgG and IgM, and cardiolipin antibody IgA and IgG. Epstein-Barr virus nuclear antigen was positive with an EBV viral capsid antigen (VCA) IgM titer of >160. The patient was continued on a low-dose prednisone tablet during the hospital stay and maintained on hydrocortisone tablets at discharge. He was placed on a prophylactic dose of enoxaparin and recommended to follow up with a hematologist after six weeks to restart full-dose anticoagulation, allowing enough time for the bleeding to settle. 

## Discussion

Antiphospholipid syndrome is known to be associated with adrenal insufficiency and hemorrhage [4,5]. Although the precise mechanism of adrenal infarction in APS is not fully understood, it is believed that the hypercoagulable state causes adrenal vein thrombosis, leading to hemorrhagic infarction of the adrenal gland. Infection with *Neisseria meningitidis* has been classically associated with adrenal insufficiency and hemorrhage; however, non-meningococcal organisms are being increasingly recognized. In recent years, COVID-19 infection, as well as vaccine-induced thrombocytopenia and thrombosis, have been associated with adrenal hemorrhage. [2] Cases of EBV-induced adrenalitis, hemorrhage, and insufficiency have also been reported in multiple pieces of literature [6-8]. Likewise, in patients with APS, EBV is known to increase the risk of venous thromboembolism. [9,10 ]

Acute adrenal insufficiency can manifest with variable symptoms such as abdominal pain, vomiting, fatigue, hypotension, and confusion, along with metabolic disturbances like hyponatremia, hyperkalemia, and hypoglycemia [11]. Most unilateral AHs are not associated with adrenal insufficiency and do not require steroid treatment. In our case, the patient did not initially have a classical presentation of hypotension; this could be explained by the marked pain causing sympathetic activation, leading to an elevation of blood pressure in the short term. However, in bilateral cases, glucocorticoid replacement should not be delayed, irrespective of symptoms, until the adequate production of cortisol is confirmed. If there is ongoing bleeding and anemia unresponsive to transfusion, endovascular embolization of the adrenal arteries or laparotomy should be considered [12]. The patient should have repeat imaging after three months to ensure the resolution of the hematoma as well as rule out an adrenal mass. Subsequently, repeat imaging can be done at three- to six-monthly intervals [13]. Additionally, early morning serum cortisol levels should be checked every three to six months for the first one to two years after the diagnosis of AH.

## Conclusions

In patients who experience sudden-onset abdominal pain and have an underlying coagulation disorder, AH should be suspected. While triggers can be idiopathic, trauma-related, infectious, or postoperative, symptoms are highly variable. There is increasing evidence that different bacteria and viruses have been found to precipitate adrenal insufficiency in predisposed individuals. Epstein-Barr virus is known to increase the risk of thrombosis in patients with APS. In this case, we want to highlight that EBV might be an underrecognized and underreported culprit precipitating AH in a coagulopathic patient. Prompt recognition and treatment are necessary to prevent adverse outcomes. Patients can have variable degrees of adrenal insufficiency; hence, steroid replacement and regular biochemical as well as radiological monitoring are essential. 
